# Mortality induced by PM_2.5_ exposure following the 1783 Laki eruption using reconstructed meteorological fields

**DOI:** 10.1038/s41598-018-34228-7

**Published:** 2018-10-26

**Authors:** Y. Balkanski, L. Menut, E. Garnier, R. Wang, N. Evangeliou, S. Jourdain, C. Eschstruth, M. Vrac, P. Yiou

**Affiliations:** 1 Laboratoire des Sciences du Climat et de l’Environnement, UMR 8212 CEA-CNRS-UVSQ-UPSaclay, Gif-sur-Yvette Cedex, France; 2 Laboratoire de Météorologie Dynamique, UMR8539 ENS-X-UPMC, Palaiseau, France; 3 UMR 6249 CNRS Chrono-Environnement, Université de Franche-Comté, Besançon, France; 4 Department of Environmental Science and Engineering, Fudan University, Shanghai, China; 5 Norwegian Institute for Air Research (NILU), Kjeller, Norway; 6 Météo-France, DClim/DEC, Toulouse, France

## Abstract

The 1783–1784 Laki eruption provides a natural experiment to evaluate the performance of chemistry-transport models in predicting the health impact of air particulate pollution. There are few existing daily meteorological observations during the second part of the 18^th^ century. Hence, creating reasonable climatological conditions for such events constitutes a major challenge. We reconstructed meteorological fields for the period 1783–1784 based on a technique of analogues described in the Methods. Using these fields and including detailed chemistry we describe the concentrations of sulphur (SO_2_/SO_4_) that prevail over the North Atlantic, the adjoining seas and Western Europe during these 2 years. To evaluate the model, we analyse these results through the prism of two datasets contemporary to the Laki period: • The date of the first appearance of ‘dry fogs’ over Europe, • The excess mortality recorded in French parishes over the period June–September 1783. The sequence of appearances of the dry fogs is reproduced with a very-high degree of agreement to the first dataset. High concentrations of SO_2_/SO_4_ are simulated in June 1783 that coincide with a rapid rise of the number of deceased in French parishes records. We show that only a small part of the deceased of the summer of 1783 can be explained by the present-day relationships between PM2.5 and relative risk. The implication of this result is that other external factors such as the particularly warm summer of 1783, and the lack of health care at the time, must have contributed to the sharp increase in mortality over France recorded from June to September 1783.

## Introduction

In 1783 the Laki fissure system that is part of the Grimsvötn volcanic system^1^, southwest of the Vatnajokull glacier in Iceland, produced one of the largest lava flow eruptions in historic times. About 15 cubic kilometers of basaltic magma was erupted from a 27 km long fissure composed of 10 smaller ones that had formed from June 1783 to February 1784^2^. The resulting lava flow flooded an area of 565 square kilometers and produced a large number of scoria and tuff cones along the fissure. The summer of 1783 was very unusual over Western Europe in several ways. First, since high occurrences of dry-fog associated with a strong smell of sulphur are reported in many locations over Europe^3^. Secondly, observers reported that for several days to weeks the sun was constantly dim as a consequence of this pervasive fog^3^. Third, as a consequence of unusual synoptic situations for the summer period, the surface temperature was on average 1 to 3 °C warmer than the mean of 1970–2000 period^4^. Considering the special climatological conditions, we can expect that the meteorological fields of years following the Laki volcanic eruptions can be different from normal years, which should affect the chemical processing and transport of air pollutants produced from the eruptions^5^. The purpose of our study is to reconstruct the meteorological fields that is closer to the conditions that prevailed in 1783 and 1784.

**Figure 1:**
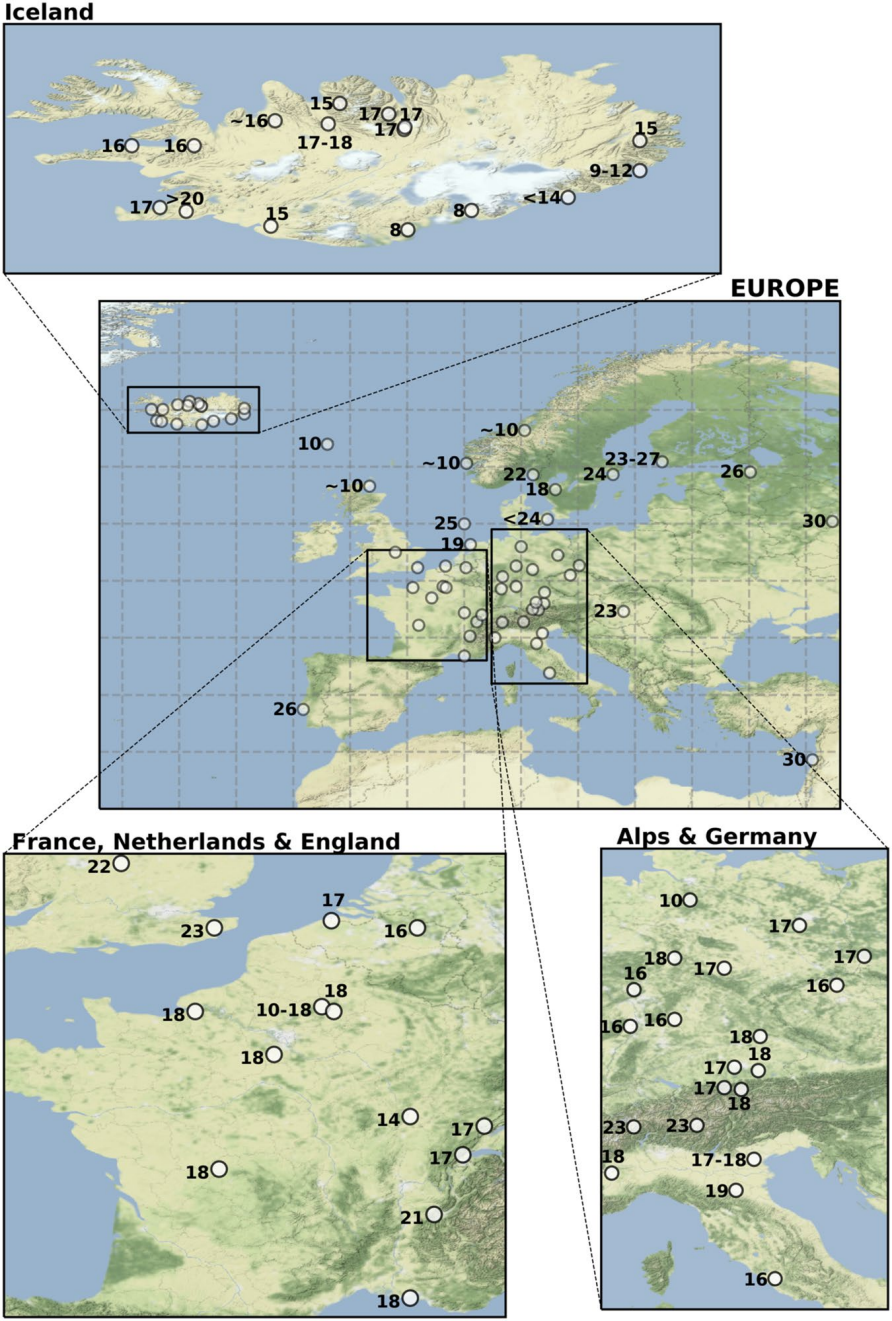
Days of June 1783 when the first manifestations of the Laki volcano are reported (After Thordarson and Self ^3^).

The first dry fogs were reported over Western Europe between June 16^th^ and June 18^th^ (Fig. 1), by the end of month of June, virtually all countries in Central and Western Europe had reported such occurences^3^. Concurrently, damages to the vegetation caused by acid rain were also reported. During peak episodes of the event, large portions of Iceland were covered by fine volcanic ash and dry fogs (gases and aerosols). The release of gas during the eruption produced a dry fog over Iceland and parts of Europe. People in Europe complained of headaches, respiratory problems and asthma attacks. In addition, 60% of the Icelandic grazing livestock died either by starvation of by fluorine which is linked to excessive ingestion of fluor emitted from the volcano^3^. It is estimated that within three years following the first eruption, 20% of the population of Iceland deceased either from their exposure to emitted gases, from being on the path of the lava flow or as a result of the famine that ensued^6,7^. The French naturalist Mourgue de Montredon was the first of several observers to correctly link the dry fog seen over Europe to the Laki fissure eruption in Iceland^8^. A few months later, Benjamin Franklin who was ambassador to France at the time, also proposed the Laki eruption was the cause for these observations over Western Europe^9^.

Climate, with its cold and warm spells, has been recognized to be a cause for excess mortality. The recent, particularly warm, summer of 2003 over Western Europe and particularly over France is one of the most striking contemporary examples^10^ by the number of time temperature records were broken in August, and also by the exceptional duration of the blocking weather pattern, that subsisted from June to August. The first reports that linked climate and public health date back to the 18^th^ century. Death certificates, extracted from catholic parish registers, have been an invaluable source to study mortality^11,12^. They have also helped studies on how death rates are influenced by infectious diseases, heat and cold waves, airborne chemicals, food shortages and social upheavals^10,13,14^. Fine particles are known for their adverse effects on human health when people are exposed to high concentrations. This has led European Union to make recommendations for PM2.5 (particles of diameter smaller than 2.5 μm) concentrations not to exceed 20 μg m^−3^ as a daily mean.

Relatively few studies focused on the role played by the Laki injection on increasing sulphur species concentrations (SO_2_ and SO_4_)^5,15,16^ on air quality, on the overall perturbation of the atmospheric sulphur cycle and on health. Even less effort was devoted to examine if these reconstructed concentrations fields could explain the unprecedented rise in death rates in the months immediately following the first Laki eruption. Noteworthy is the reconstruction of elevated SO_2_ and SO_4_ concentrations from the Laki eruption in the work of Chenet *et al*.^15^. These authors simulated sulphur as a passive aerosol tracer, 20% of which is injected at 5 km altitude while the remaining 80% are injected at 10 km. The study from these authors shows that, although the meteorological fields used are not corresponding to those in 1783, the main transport path for gases and aerosols in the weeks following the initial June 8^th^ 1783 eruption is from Iceland to western Europe.

Using a detailed sulphur chemistry in a 3D Lagrangian chemical transport model, Stevenson *et al*.^16^ tried to contrast whether SO_2_ or SO_4_ were the dominant species with regards to environmental effects of the Laki eruption. Their conclusion is that SO_2_ gas dominated sulphur deposition compared to SO_4_. The model allows to discuss the zonal mean spread of the sulphur deposition but not its entire 3-dimensional pattern. Hence, the authors could not test whether the episodes of transport arriving over Europe corresponded with the timing of the first dry fogs. A contemporaneous paper from Highwood *et al*.^17^ assessed the climate effect for such an eruption. These authors, using the SO_4_ fields from Stevenson *et al*.^16^ computed a maximum monthly mean radiative forcing in response to the series of eruptions of −5.5 W m^−2^, corresponding to a Northern Hemisphere mean temperature anomaly of 0.21 K for the whole year of 1783.

More recently, Schmidt *et al*.^5^ studied the impact of a Laki-type eruption would have on mortality in Europe if it happened in present day conditions. The main conclusions of this work were that the increase in mortality from cardiopulmonary conditions would amount to 8.3 to 8.6% over the following European countries: The Netherlands, Belgium, United Kingdom, Ireland, Germany and France. This, in turn, would cause from 96,000 to 140,000 additional deaths over Europe when using the relationship based upon the exposure-response curves of PM2.5 in the US cohorts^18^.

There are two main purposes in this paper. First, we aim to test our ability to reproduce the sequence of events observed in Europe in the first few months consecutive to ten main eruptions of 1783 starting with the one on June 8^th^ 1783^2^ (Table S1). In particular, we examine in this work the sequence of apparition of dry fogs across a path from Iceland to western Europe and compare it to predictions from a global aerosol climate chemistry-transport model nudged with reconstructed winds. The wind fields are based upon the reconstruction of sea-level pressures from measurements in 1783^19^. These reconstructed fields are obtained using a method of analogues described below. Second, we aim to model the SO_2_/SO_4_ concentrations fields during this period and test whether SO_2_ toxicity or PM (particulate matter) excess concentrations could explain the peak in mortality reported in the deaths registers from French parishes in the summer of 1783^12^.

## Results

### Simulation of the pollutant transport following the Laki eruptions

The serie of ten eruptions over months from June to October 1783 were grouped into three phases according to Thordarson & Self ^2^: The first phase consists of three separate eruptions that occurred from June 8^th^ to 15^th^. These 3 eruptions alone are the most violent and efficient in producing sulphur. The second phase from June 25^th^ to September 1^st^ 1783 includes four large eruptions that are more spread in time than during the first phase. The third phase, from September 7^th^ to October 9^th^, is characterized by less intense eruptions that are even more spread in time. The most remarkable manifestation of the Laki eruption that was reported over Europe are the so-called dry fogs. Thordarson and Self ^3^ synthesized the reports that contained the dates of the first appearances of these dry fogs over Europe that correspond to the first manifestations of the volcano. Matching these days with the time of arrival of SO_2_ over these regions, constitutes a stringent test of whether we are able to represent the SO_2_ distribution and its propagation from Southern Iceland to continental Europe and then to Great Britain. We chose the following five stringent criteria to analyze whether the progression of the SO_2_ linked to the apparition of the first manifestation of the Laki is well captured by the model (see also Fig. 1).

**Figure 2:**
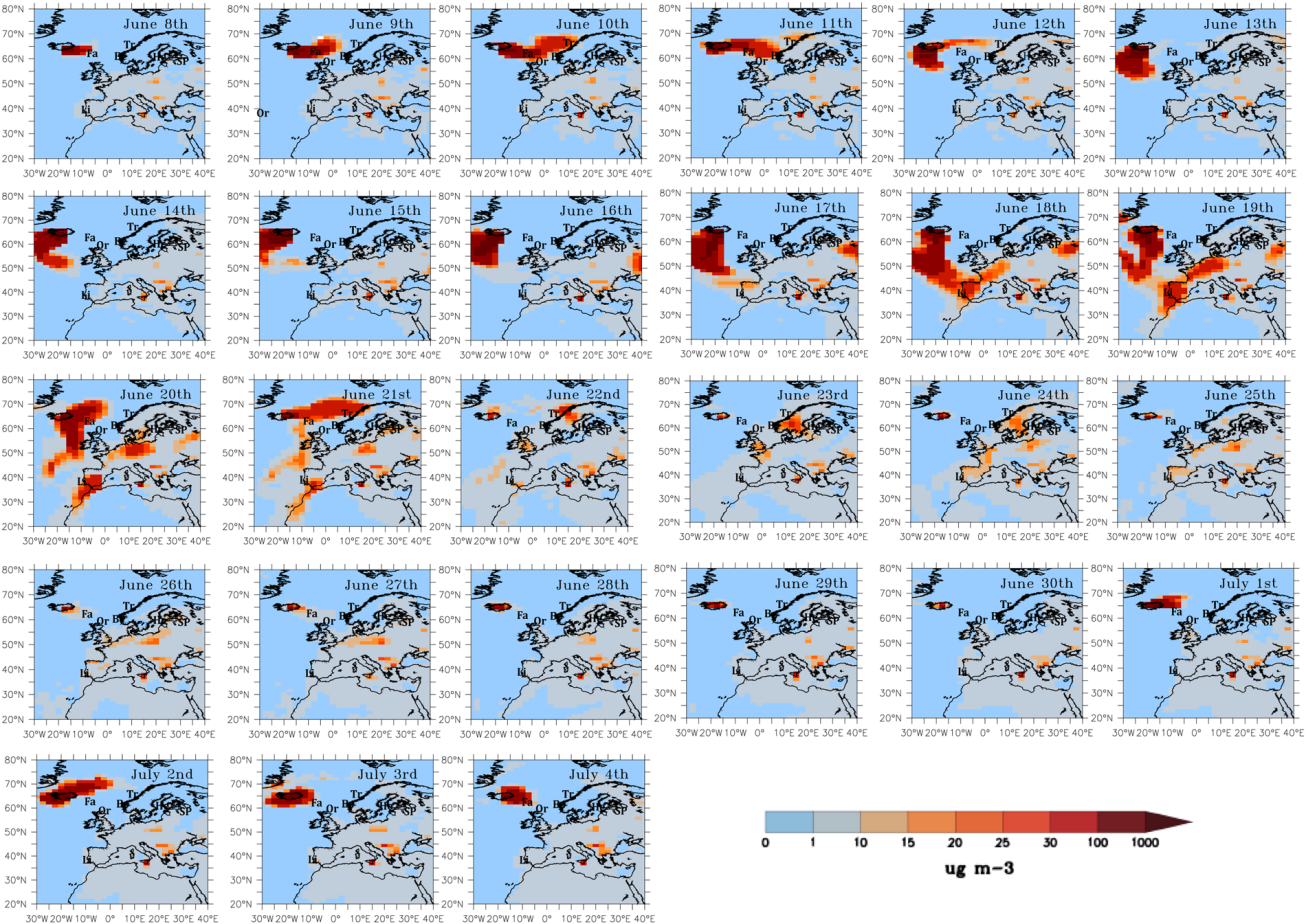
Daily averaged SO_2_ concentrations (μg m^−3^) from 0 to 5.6 km when dry fogs were first reported over Europe for the period from June 8^th^ to July 4^th^ 1783. Locations discussed in the text are indicated as: “Fa” for the Faroe Islands; “Or” for the Orkney Islands, at the most northeastern tip of Scotland; “Tr” for Trondheim, Norway; “Be” for Bergen, Norway; “Li” for Lisbon, Portugal, “He” for Helsinski, Finland; and “SP” for St Petersburg, Russia.

On June 8^th^, the cloud of cinder and gases that emanates from the eruption is reported to reach the coast in two places towards the South and South-East ends of Iceland according to Thordarson and Self ^3^. The global simulation reproduces exactly these conditions on the days of the first eruptions as the transport is first South and then East, while SO_2_ moves away from Iceland (Fig. 2).

On June 10^th^, two days after the first eruption, its first manifestations were reported over the Faroe Islands, on the very northeastern tip of Scotland, and on two locations over Norway: Bergen and Trondheim^3^. The transport pattern produced from the method of analogues perfectly captures the extent of the SO_2_ clouds over these locations. SO_2_ concentrations exceed 30 μg m^−3^ off the coast of Norway, and the distribution of SO_2_ concentrations peaked over the Faroe Islands extending to the northeastern tip of Scotland (Fig. 2). Until June 12^th^, the plume of SO_2_ was confined North of 55°N. From June 13^th^ to 16^th^ the SO_2_ plume progressed southwards over the Central Atlantic reaching Ireland and Brittany (Western France).

The first reports of dry fogs and sulphur smell over France, Italy, Switzerland and Prussia occurred on June 17 and 18^th^ ^3^. These are the days when the plume spread over Western Europe invading France and Prussia (Fig. 2), as a low pressure system was positioned over the British Isles (Supplementary Fig. S1).

Starting on June 21^st^ and up to June 23^rd^, reports indicate very intense manifestations of the cloud that passed from West to East over South England. A large tongue of SO_2_ concentration between 10 and 25 μg m^−3^ extended from Iceland to the Azores on June 21^st^. Then, it travelled eastwards to reach Western England on June 22^nd^ and Eastern England on June 23^rd^ (Fig. 2). During these days, very heavy fogs were reported over England.

On June 26^th^, dry fogs caused by the Laki eruptions were reported over Lisbon, Portugal and St Petersburg, Russia. Concentrations of SO_2_ in excess of 30 μg m^−3^ are simulated over Portugal and Spain on June 18^th^, 19^th^ and 20^th^, and concentrations between 10 and 15 μg m^−3^ on June 24^th^ and 25^th^. On June 18^th^, 19^th^ and 20^th^, high SO_2_ concentrations can be observed over Spain, Portugal, western France, and most of northern Europe. We simulate SO_2_ concentrations between 10 and 15 μg m^−3^ over St Petersburg on June 21^st^ and June 26^th^. Reports from Lisbon, Helsinski and St. Petersburg indicate the fog formation, which was later attributed to the eruptions of Laki (Fig. 2).

**Figure 3:**
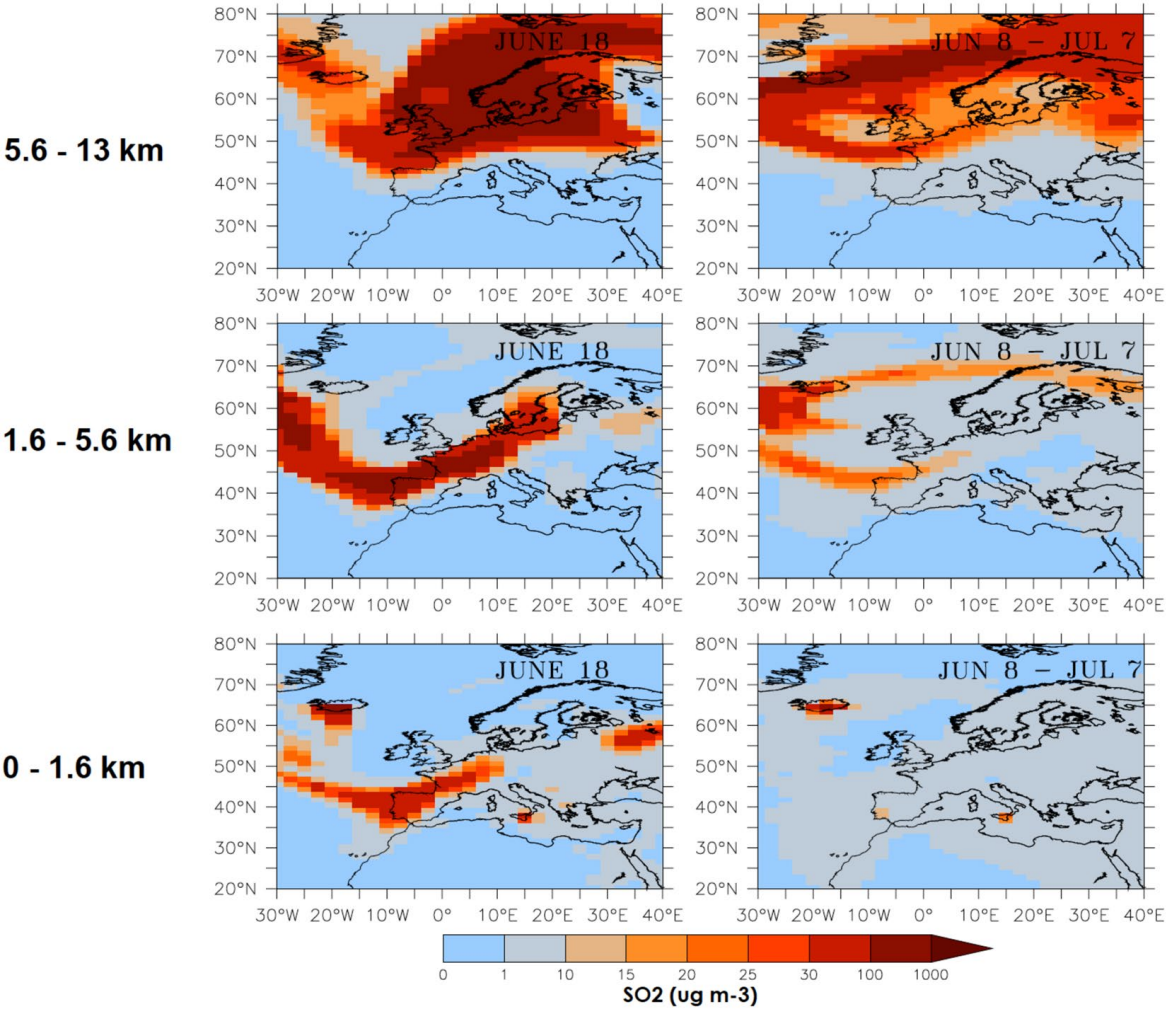
Left column: daily mean (June 18^th^ 1783) and right column: monthly mean SO_2_ from June 8^th^ to July 7^th^ concentrations (μg m^−3^) simulated in the boundary layer, free troposphere and mid- to high troposphere Top row: from 5.6 to 13 km, middle row: from 1.6 to 5.6 km and bottom row: from the surface to 1.6 km.

**Figure 4:**
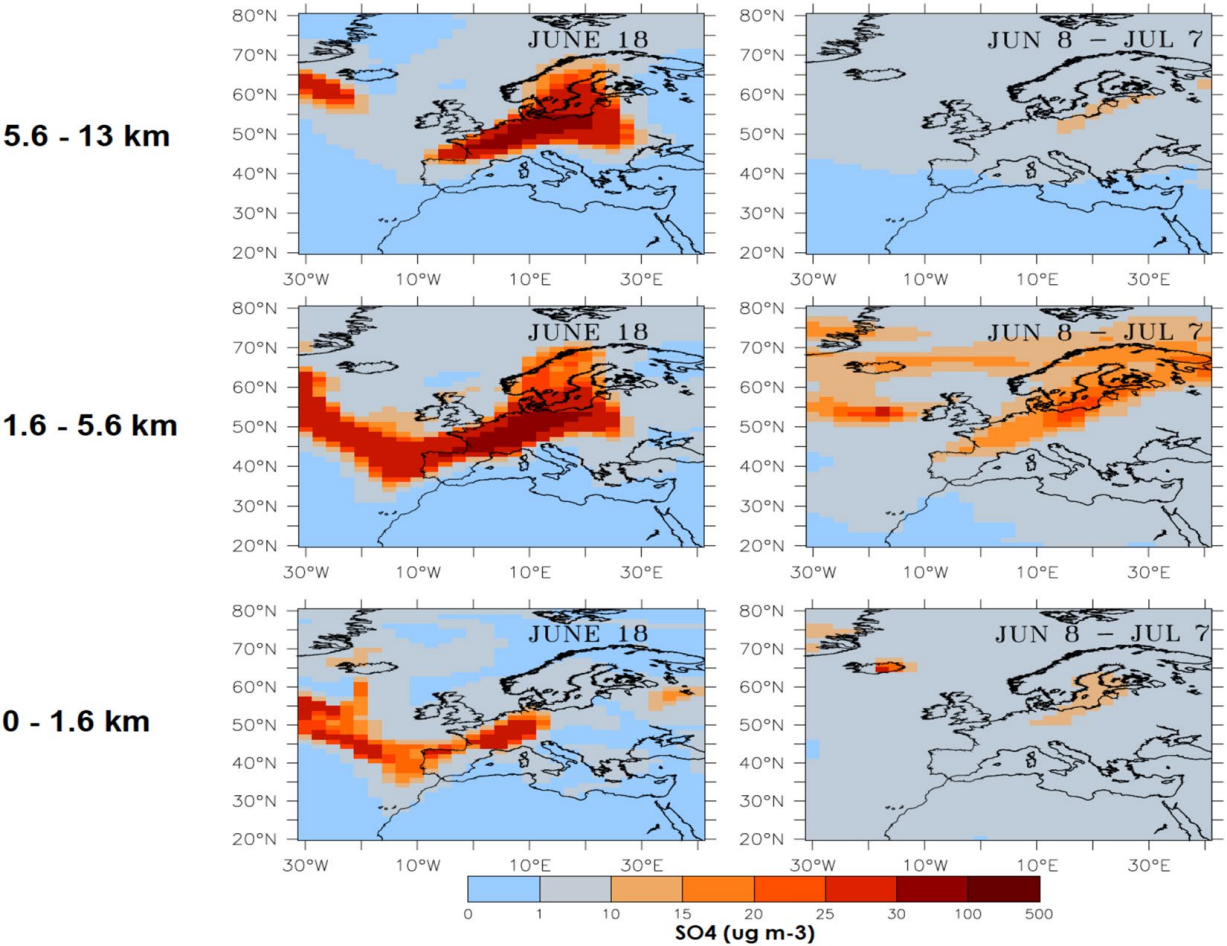
Left column: daily mean (June 18^th^ 1783) and right column: monthly mean SO_4_ from June 8^th^ to July 7^th^ concentrations (μg m^−3^) simulated in the boundary layer, free troposphere and mid- to high troposphere. Top row: from 5.6 to 13 km, middle row: from 1.6 to 5.6 km and bottom row: from the surface to 1.6 km.

We focus our analysis on the study of two periods with an intense transport of SO_2_ and SO_4_ from Iceland towards Europe’s western boundary. Figures 3 and 4 illustrate how SO_2_ and SO_4_ respectively enter Europe’s boundary layer (0–1.6 km), lower to mid troposphere (1.6–5.6 km) and mid to high troposphere (5.6–13 km). We chose two different periods illustrating, first when dry fog manifestations were reported (June 18), and secondly, for a one-month period when the Laki eruptions intensity is maximum (June 8–July 7). Figure 3 shows that SO_2_ concentrations in the boundary layer over Southwestern France reach up to 25 μg m^−3^ for the June 18 period, and are much more elevated (in excess of 100 μg m^−3^) in the free troposphere. Figure 4 illustrates PM2.5 that is composed of SO_4_ produced by SO_2_ in-cloud oxidation and by the oxidation of SO_2_ with OH on short timescales from hours to days. It is worth noting the differences between SO_2_ and PM2.5 concentrations by comparing Figs 3 and 4. On June 18, PM2.5 boundary layer concentrations range between 25 and 30 μg m^−3^ over a broad band that goes from SW France to the Netherlands. PM2.5 concentrations are much higher (between 30 and 100 μg m^−3^) in the lower troposphere and over a larger region that extends from SW France to Prussia. The monthly SO_2_ concentrations both in the boundary layer and in the free troposphere from June 8^th^ to July 7^th^ are less elevated over Western Europe than during the June 18^th^ period. The large PM2.5 concentrations are found in regions corresponding to maximum SO_2_ concentrations. Monthly mean concentrations of SO_2_ and PM2.5 (SO_4_ concentrations) from June 8 to July 7, are both more elevated in the low troposphere (from 1.6 to 5.6 km) than in the boundary layer (from the surface to 1.6 km).

We discuss below the relative risk, related to excess mortality when compared to mean mortality over the 16-year period: 1774–1789, from such high concentrations.

### Health impact of PM2.5 exposure by SO_2_ emissions from Laki volcanic eruptions

**Figure 5:**
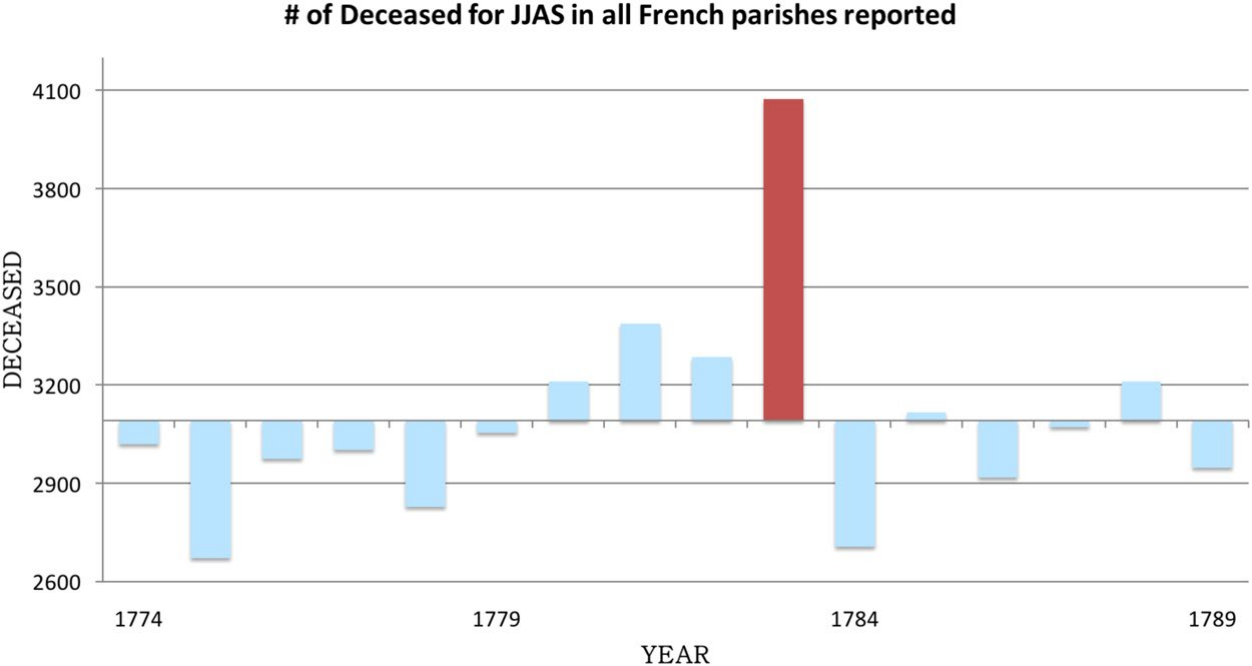
Deviation from the mean number of JJAS deceased in France over all the parishes listed on Table 2. The column in red indicates the summer of 1783 that is discussed in the text.

Prior to the work of Garnier^12^ on deaths statistics over all regions of France, the records of only a few parishes from three different provinces had been compiled^20^. These records from 1782 to 1784 came from three French regions: Loiret, Seine-Maritime and Eure-et-Loire. Over these regions, the mortality over the period August–October 1783 is 48% above the mean of the 3 years studied^20^. Mortality over England for the 1783–1784 period has been better documented than the one in France. Witham and Oppenheimer^11^ discuss the excess mortality from August to September 1783 as well as a peak in January–February 1784 and estimate at 20,000 extra deaths the toll for England of the consequences of the Laki, together with the abnormally warm summer of 1783. Moreover, winter 1783/1784 showed a pronounced increase in the deceased individuals comparing to other years. This translates into a number of burials between August 1783 and May 1784 that is 25% greater than the average of the 3 years. We present deaths statistics extracted from the registers of death certificates compiled by Garnier^12^ for parishes that are located over all French regions, lasting from January 1774 December 1789. Garnier^12^ chose parishes for which the registers of death certificates were recorded and quality-checked. For this analysis, all reported deaths were tallied over June-July-August-September (JJAS) of 1783. Supplementary Fig. S2 shows the locations of the parishes from Table 1 for which the anomalies in death rates of each JJAS period from 1774 to 1789 were analysed. The ensemble of French parishes reported here recorded 4193 individuals deceased in JJAS 1783, a 32% higher number than the average of 3092 deceased for the period 1774–1789 (Fig. 5). The second largest anomaly for the period occurs in 1781, with only 10% higher death number (3187) than the 16-year average. The increase in mortality in 1779 is a consequence of a dysentery epidemic that took place in Brittany (Western France)^14^. Hence, the deviation from the mean of the summer 1783 anomaly of deceased is 3 times greater than any other year of that period. We next grouped geographically the numbers of deceased reported over the following regions: Northern, Eastern, Southern, Central and Western France. It should be noted that the population and hence the number of deaths reported is not homogeneous across regions. For all the parishes reported by Garnier^12^, 40.3, 15.3, 33.8, 0.9 and 9.7% of the reported deceased respectively belonged to Northern, Eastern, Southern, Central and Western France. Supplementary Fig. S3 illustrates that for 3 out of 5 regions, JJAS 1783 is the summer period of the 16 years with the most deceased. Over Western France, it represents the second period after 1779 with the most deceased and the fourth over Eastern France.

**Table 1:** Days with SO_2_ concentrations greater than 125 μg.m^−3^ at all cities where statistics for deceased have been reported.

Locations	Nb of days SO_2_ > 125 μg.m^−3^ STP (surface –1.6 km)	Min/Mean/Max SO_2_ conc. > 125 μg.m^−3^ (surface – 1.6 km)	% Increase RR* (surface – 1.6 km)
Laki, Iceland	55	125.3/186.50/296.0	13–30
Faroe Islands	0	NA	
Orkney Islands	0	NA	
All French sites: (Dunkerque- Lille Cambray- St-Malo- Suillé-le-Gravellais- Clisson- Poitiers- La Rochelle- Paris - Naveil- Laon- Nancy- Lyon- Montpellier)	0	NA	

*Relative risk (RR) is computed from the relationship RR = 1.01 per 10 μg/m^3^ SO_2_ (Ko *et al*.^21^).

There are indications that the increase in mortality during the summer months immediately following the Laki eruption is not caused by scarcity of food. There are no reports of food shortages in Western Europe for 1783. In addition, the registers of deceased include information concerning the profession of the individuals. When hunger is a cause for mortality, its occurrence is biased towards the poorest individuals. Garnier^12^ showed that the mortality during the summer of 1783 did not affect more severely individuals from families having low-paid jobs compared to more prosperous ones. No epidemics of flu, smallpox and cholera occurred over this year^16^. As a consequence of unusual synoptic situations for the summer period, the surface temperature in 1783 was on average 1 to 3 °C warmer than the mean of 1970–2000 period^4^. Hence, we may conclude that most abnormal deaths for that summer would be the consequence of the warm spell associated with the very intense concentrations associated with the dry-fog that prevailed.

Table 1 illustrates the simulated number of days when SO_2_ concentrations exceeded the limit of 125 μg m^−3^ over Iceland, over the Faroe Islands and over French cities from July to September 1783. Ko *et al*.^21^ describe the value of 125 μg m^−3^ of SO_2_ as a threshold after which long exposure of several hours would cause an excess rate of death of 1% per increment of 10 μg m^−3^ of SO_2_. Based upon this work, we compute that the excess SO_2_ from the Laki eruption increased from 13 to 30% the death rate of the Icelandic population (Table 1). SO_2_ concentrations were below 125 μg m^−3^ on the Faroe Islands or over western Europe and hence did not cause an excess in death rate.

Table 2 illustrates the simulated number of days when SO_4_ concentrations exceeded that limit of 20 μg m^−3^ over French cities from July to September 1783. Since PM2.5 concentrations from the Laki can be detrimental to health, we next assess whether the excess mortality observed in JJAS 1783 could be explained solely by PM2.5 concentrations. Environments with elevated PM2.5 are known to be a cause for risk. The study of Pope *et al*.^18^, which followed up cohorts from American cities for a length of time of over 15 years, was the basis for building relationships between PM2.5 concentrations and excessive mortality. Schmidt *et al*.^5^ expressed the relative risk, *RR*, as a function of the difference in PM2.5 caused by a Laki-type eruption extending to high PM2.5 concentrations the short-term mortality relationship:

1 $$RR={e}^{[\gamma ({X}_{Pd}-{X}_{Cd})]}$$

where *X*_*Pd*_ is the PM2.5 concentrations of the period of the Laki eruption, and *X*_*Cd*,_ is the daily mean PM2.5 concentrations over the JJAS periods for 1774–1789, and γ is the exposure-response coefficient.

**Table 2:** Number of days with PM2.5 greater than 20 μg.m^−3^ at all cities where statistics for deceased have been reported.

City	Number of days in JJAS with PM2.5 > 20 μg.m^−3^	Min./Mean/Max. daily averaged PM2.5 (μg.m^−3^)	Increase risk of mortality from Integrated Exposure-Response (IER) model (%)
Laki, Iceland	70	0.0/34.0/148.3	59.6
Faroe Islands	0	0.0/2.5/19.2	0.9
Orkney Island	0	0.0/2.1/17.5	0.5
Dunkerque	2	0.0/2.5/24.4	1.5
Lille	2	0.0/2.5/24.4	1.5
Cambray	2	0.0/2.5/24.4	1.5
St Malo	0	0.0 1.9/16.3	0.4
Ruillé-le-Gravellais	2	0.0/2.4/34.2	1.6
Clisson	1	0.0/2.2/32.8	1.0
Poitiers	3	0.0/2.7/43.5	2.1
La Rochelle	3	0.0/2.8/42.7	2.3
Paris-Créteil	3	0.0/2.7/34.8	2.1
Naveil	2	0.0/2.4/34.2	1.6
Laon	3	0.0/2.7/34.8	2.1
Nancy	3	0.0/3.1/70.6	3.0
Lyon	2	0.0/2.6/48.7	1.7
Montpellier	2	0.0/2.2/12.9	0.2

As pointed out by Schmidt *et al*.^5^, the results of the cohort study from which the relative risk is computed in Eq. (1) is a linear exposure function for a range of PM2.5 concentrations from 0 to 40 μg m^−3^. Hence, this relationship does not necessarily apply to PM2.5 concentrations higher than 40 μg m^−3^. Years after the work of Pope *et al*.^18^, further studies that included second-hand smoking and indoor pollution studies revealed that excess mortality is non-linear. To account for PM2.5 concentrations between 7 to 600 μg m^−3^, Burnett *et al*.^22^ integrated the information from different studies including household air pollution, second hand smoking and ambient air pollution. This range of concentrations allows for studying the intense episodes following the Laki eruption. We next apply the relationship formulated using an integrated exposure-response model (IER) that covers a PM2.5 range from 7 to 600 μg m^−3^. This range includes the elevated hourly mean concentrations that can be recorded over the most polluted cities in Asia, as well as the daily mean concentrations that we calculate over continental Europe from the successive Laki eruptions. The study of Burnett *et al*.^22^ also provides confidence intervals for predicted lower and higher values of excess mortality from the Integrated Exposure-Response (IER) model.

Whereas the predicted mortality calculated using the linear model underestimates the reported increase of deceased individuals of the summer 1783 by one order of magnitude (Table 2), the relationship derived from the IER model suggests that this excess mortality is linked to 1 to 3 events of PM2.5 concentrations (>20 μg m^−3^) that occurred from June 18^th^ to September 30^th^ 1783. Over France, the IER model suggests that the abnormally large mortality increase is not solely due to the impact from the volcano. Instead, the high mortality rates recorded over France could be accounted for by a combination of frail people, of the effects of the heat wave that occurred that summer, in combination with the dry fogs and the absence of health care for people with respiratory conditions.

It should be noted that we cannot exclude other possibilities that can explain the underestimation of the mortality predicted by the linear model or the IER model. As a key parameter in these health-risk models, the exposure-response coefficient (γ) is subject to high uncertainty, and can vary by region and time. For example, Pope *et al*.^23^ suggests that the estimated relative risk corresponding to an exposure of PM2.5 concentrations of 40 μg m^−3^ can vary from 1.05 (an increase in mortality by 5%) to >1.50 (an increase in mortality by >50%), due to variance of the input (γ). Keep in mind that the coefficient (γ) depends on how sensitive people are to the PM2.5 exposure. It is very likely that people are more vulnerable to air pollution (higher γ) since health care was much less developed in 1780s than present day.

### Summary of results

We reconstructed 6-hourly meteorological fields for 2 years: 1783 and 1784 using the method of analogues^4,24^ applied to the 40 years ERAI-reanalysis^25^. Two historical datasets from the summer of 1783 were analyzed to assess:

1. Whether the reconstructed meteorological fields led to better resolve the distribution of sulphur species emitted by the successive volcanic eruptions from June 1783 to February 1784;
2. If the relationships derived from health studies on relative risk based upon daily mean PM2.5 concentrations could explain the surge in mortality over France in JJAS 1783.

On both counts, this study leads to novel results that help us better understand the fate of pollutants and their effect on excess death rate (excess death rate is computed relative to the 16-years period from 1774 to 1789) in the case of extreme eruptions.

We can explain several of the main features of the apparitions of the first manifestations of the volcano over Western Europe:

- On June 10^th^ 1783 over the Faroe Island, Aberdeen, the northeastern tip of Scotland and Bergen and Florø, Norway.
- on June 17 and 18^th^ reaching France and Germany Northern Italy,
- on June 21 to 23: Reports that the cloud over South and Southwestern England.

There are limitations to the modelling shown here: namely, the elevated SO_2_ concentrations before June 21 over South England and before June 26 over Lisbon have not been reported. A significant part of these limitations could come from going from a limited set of stations to a gridded product as we apply the technique of analogues.

We have shown that this work constitutes a step forward in capturing the majority of observed synoptic situations compared to previous published studies.

The integrated exposure-response model from Burnett *et al*.^22^ that aggregates results from indoor pollution with those from active smoking concurs with the conclusion previously drawn that the majority of the excess mortality that occurred in the summer of 1783 over France is not solely the result of the high concentrations of SO_2_ emitted by the Laki volcano. Since there were no famines at the time^13^, the very significant increase in mortality over the summer of 1783 must have been brought about by a combination of high SO_2_ and PM2.5, extreme heat during the summer of 1783, the fragility of the people, and the absence of health care^26^.

## Methods

### An aerosol climate chemistry-transport model LMDZ-INCA

The aerosol module INCA (Interactions between Aerosols and Chemistry) is coupled to the general circulation model LMDz, developed at the Laboratoire de Météorologie Dynamique in Paris. Hauglustaine *et al*.^27^ describe the gas phase chemistry included in INCA. Aerosols and gases are treated in the same code to ensure coherence between gas phase chemistry and aerosol dynamics as well as possible interactions between gases and aerosol particles. The simulations employed in this study were achieved using a horizontal resolution of 2.5° by longitude and 1.27° by latitude and the vertical direction was discretized into 39 layers from the surface to 80 km using terrain-following coordinates. The simulation lasted 2 years and started on January 1^st^ 1783. The first period from January 1^st^ to June 8^th^ serves as a spin-up to the model. The following four basic properties matter for the sulphate that is present in the form of aerosol: size, chemical composition, hygroscopicity (HG) and mixing state of the particles. The major sources for aerosol sulphate, other than sea salt sulphate, are DMS and SO_2_ and to a minor extent H_2_S. The model simulations include emission fluxes of DMS and of SO_2_ for the pre-industrial period (1750) based upon the dataset published by Dentener *et al*.^28^. These sources of SO_2_ include emissions from wild-land fire, biofuel, domestic sectors and volcanic emissions. Natural volcanic emissions amount to 2.0 TgS/yr of explosive emissions, and 1.9 TgS/yr of continuous emissions^28^. DMS emissions in INCA are computed from maps of monthly sea surface concentrations of DMS from Kettle and Andreae^29^, and the actual air-sea-gas exchange coefficient, taking into account sea water temperature and wind speed^30^. We assume that DMS emissions from the ocean were the same in 1783 than at present. The chemical transformation of the gaseous sulphur species requires oxidants either in the gas-phase or in the liquid-phase. The version of the sulphur chemistry implemented here is similar to that of Boucher *et al*.^31^. At each time step of the model, all reactive species included are updated according to transport processes, sources and sinks. Sulphur chemistry in INCA is part of the dynamic chemistry scheme that has been evaluated and compared to other models within the AeroCom (Aerosol Comparisons between Observations and Models) initiative^32–34^. DMS and its product DMSO are oxidised using the actual concentrations of OH and NO_3_. SO_2_ is transformed to sulphate by H_2_O_2_ and O_3_ in cloud liquid water. The formation of sulphate is limited by the acidity formed in the oxidation process in the cloud droplets. SO_2_ is also oxidised in the gas-phase. Gaseous H_2_S and aerosol methane sulphonic acid (MSA) are also included as minor species of the sulphur cycle. We account for the hygroscopicity of the particle following a relationship derived from measurements reported in Swietlicki *et al*.^35^ for coexisting hydrophobic and hydrophilic particles.

### Reconstruction of the meteorological fields using a method of analogues

Until now, reconstructions of SO_2_/SO_4_ and H_2_SO_4_ atmospheric fields consecutive to the successive Laki eruptions have been uncertain in great part due to the absence of meteorological fields representative of the circulation that prevailed during the time when the Laki eruption was most active. The only meteorological data that is available for that period is a daily SLP reconstruction compiled by Kington^19^. To be able to nudge the model, we need to have 3 dimensional fields of wind data with a time resolution of 6 hours. This requirement does not allow us to use directly the resulting daily sea-level pressures from Kington^19^. In practice, we applied the method of analogues to the gridded data set of sea-level pressures (SLP) from Kington^19^ that spans from January 1^st^ 1781 to November 30^th^ 1785. It is based on measurements from 70 stations over Europe with relatively good coverage over France, Prussia and Great Britain, and with fewer stations covering Iceland, Scandinavia, Spain and northern Italy. The region covered by this ensemble of stations extends from 30°W to 30°E and from 35 to 70°N^19^. Biases can be introduced when going from the set of stations to a gridded product. Details about the method and the data quality check in order to remove outliers and detect errors in dating can be found in Yiou *et al*.^4^. The geographical zone that is covered by the Kington^19^ SLP dataset and constrains the analogue search is close to the one recommended by Jézéquel *et al*.^36^ to simulate western European temperatures.

Assessing an uncertainty on the gridded SLP reconstructed is a difficult task. Yiou *et al*.^4^ compared the historical observed times series at 10 locations over Europe with the temperatures simulated when using the analogues. The score of this quantitative comparison is modest, although a qualitative comparison with historical trends^37^ is satisfactory. In the present work, we use the method of analogues to select, from the observations of SLP for 1783, an ensemble of 6-hourly 3-dimensional wind fields. To retrieve the dates that allow us reconstructing these fields, we minimize the root mean square (RMS) of the difference between ECMWF ERAI^25^ daily fields of SLP from the reanalysis, and daily-observed field of SLP for the years 1783 and 1784. For each day, the 20 best analogues are saved based upon their smallest RMS. The procedure is repeated for each day of these 2 years.

Reconstructed meteorological fields used to transport inert or chemically reactive species need not only to be continuous but one also needs to make sure that we have consistency between two juxtaposed time periods. Yiou *et al*.^24^ determined, based upon the autocorrelation with time lag, that continuity was optimal when taking a 5-day window to retain a significant correlation between observed and reconstructed fields. To have consistency between two juxtaposed time periods, we overlap the last 2 days of any given 5-day window with the 2 first days of the consecutive one, and apply a weighted fit that shifts the weight linearly from one window to the next. As the 20 best analogues are saved for each time period, and they are 120 periods of 3 days that can be used over a year with 365 days, there are 20^120^ possibilities per year reconstructed. For the present study, we chose the best analogue of each period and stored the meteorological fields associated. The analogues reconstructed by Yiou *et al*.^24^ with this technique between 1 January 1781 and 30 September 1790, show an average spatial correlation coefficient of 0.7 with the SLP fields derived by Kington^19^ for these years. This value is similar to what was found in the assessment of Yiou *et al*.^24^ on geopotential heights in reanalyses, with correlations decreasing with altitude.

### Injection height of SO_2_/SO_4_ emissions

The literature has assessed what could have been the heights of fire fountains that were a particularly prominent feature of the Laki eruption. Thordarson and Self ^2^ used the assumption that, in order to be seen from central South Iceland, these fire fountains should exceed 1350 m in height. In the case of eruption with a fissure length, *L*, that exceeds the column height that determines the maximum injection, *H*, Stothers *et al*.^38^ and Stothers^39^ proposed a relationship to derive the column height as a function of the thermal heat released per unit length:

$$H={(\frac{Q}{L})}^{0.333}$$

where Q is the rate of thermal energy released.

This simple estimation leads to an average height of injection during the 10 first days of the eruption of 4360 m corresponding to a height of 5500 m to 6000 m above sea-level once the height of the fire fountains is added.

The estimation of column heights for short fissures of circular vents that Thordarson and Self ^2^ also used leads to maximum heights of 10000 to 11000 m for the first 3 episodes, or 12000 to 13000 m a.g.l. when one accounts for the fire fountains height. Based upon these estimates, and building on the simulations of Stevenson *et al*.^16^, 30% of the SO_2_ emissions described in Table S1 are injected from surface to 1600 m, and 70% from 5600 m to 12700 m altitude a.s.l. (Table S2). A total of 63 Tg(S) or 125 Tg(SO_2_) is injected as described in Table S1, following the sequence and the daily amounts described by Stevenson *et al*.^16^ and in Thordarson and Self ^2^.

## Electronic supplementary material

Supplementary Information

## Data Availability

The datasets generated during and/or analysed during the current study are available from the corresponding author on reasonable request.
