# Supplementary material for: Mortality induced by PM2.5 exposure following the 1783 Laki eruption using reconstructed meteorological fields
Source: Sci Rep. 2018 Oct 26;8:15896. doi: 10.1038/s41598-018-34228-7 (PMC6203706; doi:10.1038/s41598-018-34228-7)
Supplement: Supplementary file 1 — Supplementary Information [file 41598_2018_34228_MOESM1_ESM.pdf]

Supplementary Information

Mortality induced by $PM_{2.5}$ exposure following the 1783 Laki eruption using reconstructed meteorological fields

Y. Balkanski[1,*], L. Menut[2], E. Garnier[3], R. Wang[4], N. Evangeliou[5], S. Jourdain[6], C. Eschstruth[1], M. Vrac[1], P. Yiou[1]

[1]Laboratoire des Sciences du Climat et de l'Environnement, CEA-CNRS-UVSQ-UPSaclay Gif-sur-Yvette Cedex, France

[2]Laboratoire de Météorologie Dynamique, CNRS-Ecole Polytechnique, Palaiseau, France

[3]UMR 6249 CNRS Chrono-Environnement, Un. de Besançon, France

[4] Department of Environmental Science and Engineering, Fudan University, Shanghai, China

[5]Norwegian Institute for Air Research (NILU), Kjeller, Norway

[6]Météo-France, DClim/DEC, Toulouse, France

* Corresponding author: Y. Balkanski (yves.balkanski@lsce.ipsl.fr)

The supplementary information is composed of 3 Tables and 3 Figures.

SUPPLEMENTARY LEGENDS.

Supplementary Table S1: Dates, mass of SO_2 injected and period number of the 1783 Laki injections.

Supplementary Table S2: Characteristics of the model layers and normalized fraction of the SO2 injected in the experiment.

Supplementary Table S3: Comparison of the reported mortality with the estimated mortality reported in the linear model of Equation 1in the text and with the integrated exposure relationship model from Burnett et al.[15].

Supplementary Figure S1: Left, position and intensity (mb) of the high and low pressures at sea-level as reported by Kington 1999[15] for 18 June 1783. Right, Closest analogue of sea-level pressure (mb) for 18 June 1783 selected by the method of analogues presented in the Methods section.

Supplementary Figure S2 **:** Cities for which parishes with registers of Baptism, Weddings and Deceased were analysed to infer the increased mortality of the summer (JJAS) 1783 relative to the 6-years mean from 1774 to 1789. The dates indicated in red point to periods of frequent fog observations.

Supplementary Figure S3: Deviation from the mean number of deceased for the period JJAS. The list of parishes is given on Table 2. The column marked in red indicates the JJAS period for 1783.The deviation (%) of the number of deceased from the mean of 15 years is indicated. Note that the different regions have unequal numbers of deceased (Table S3) that correspond to the number and size of the parishes that have been analyse. Respectively, 40, 15, 1, 10 and 34% of the deaths occurred in Northern, Eastern, Central, Western and Southern France.

Table S1: Dates, mass of SO_2 injected and period number of the 1783 Laki injections.

Date in 1783	Mass Injected ($Mt\ SO_2$)	Injection Period No Unit
8 June – 10 June	10.5	1
11 June – 13 June	17.2	2
14 June – 22 June	23.0	3
25 June – 29 June	10.3	4
11 July – 21 July	13.2	5
29 July – 09 Aug.	17.5	6
30 Aug. – 03 Sep.	11.9	7
06 Sep. – 14 Sep.	10.5	8
25 Sep. – 29 Sep.	5.9	9
26 Oct. – 31 Oct.	5.0	10
Sum injected	125.0	

Table S2: Characteristics of the model layers and normalized fraction of the SO_2 injected in the experiment. The amounts of SO_2 injected are indicated in Table S1.

Model Level	Top-Level Height (m) (m agl)	Layer Thickness (m)	Normalized fraction of SO_2 injected
18	12719	996	0.063
17	11723	1034	0.077
16	10689	1139	0.100
15	9550	1249	0.130
14	8301	1295	0.157
13	7006	1245	0.174
12	5761	1125	0
11	4636	973	0
10	3663	816	0
9	2847	668	0
8	2179	536	0
7	1643	420	0
6	1223	322	0.092
5	901	239	0.071
4	662	171	0.052
3	491	121	0.037
2	370	86	0.026
1	284	70	0.021

Table S3: Comparison of the reported mortality for the period JJAS with the estimated mortality calculated for JJAS from the linear model of Equation 1 in the text and with the estimated mortality calculated from the integrated exposure relationship model from Burnett et al.[15].

Region	Average Number of Deaths Reported from 1774 to 1789	Excess mortality IER model (%)	Excess mortality from SO_2 exposure (%)	Excess mortality reported in 1783 compared to the 1774 - 1789 period (%)
Northern France	1243	0.0 / 2.4 / 34.2	1.6	29
Western France	298	0.0 / 2.5 / 19.2	0.9	22
Central France	27	0.0 / 2.1 / 17.5	0.5	77
Eastern France	484	0.0 / 2.5 / 24.4	1.5	25
Southern France	1041	0.0 / 2.5 / 24.4	1.5	40
Total (all regions)	3092	0.0 / 2.5 / 24.4	1.5	32

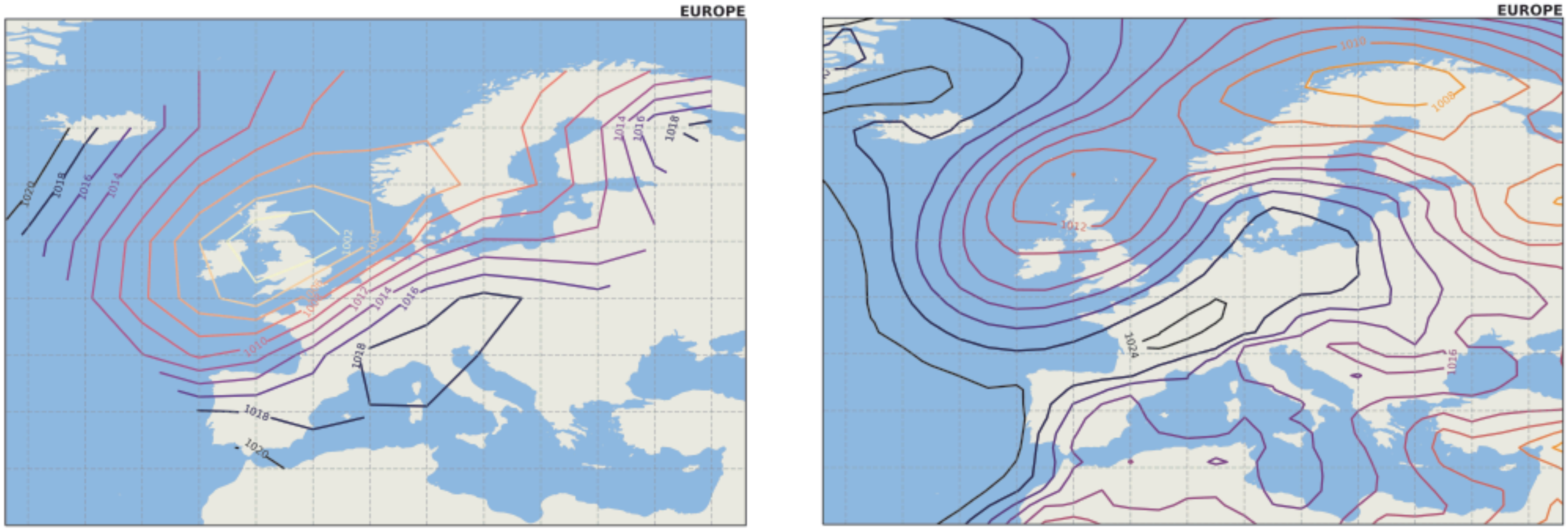

Supplmentary Figure S1: Left, position and intensity (mb) of the high and low pressures at sea-level as reported by Kington 1999[16] for 18 June 1783. Right, Closest analogue of sea-level pressure (mb) for 18 June 1783 selected by the method of analogues presented in the Methods section.

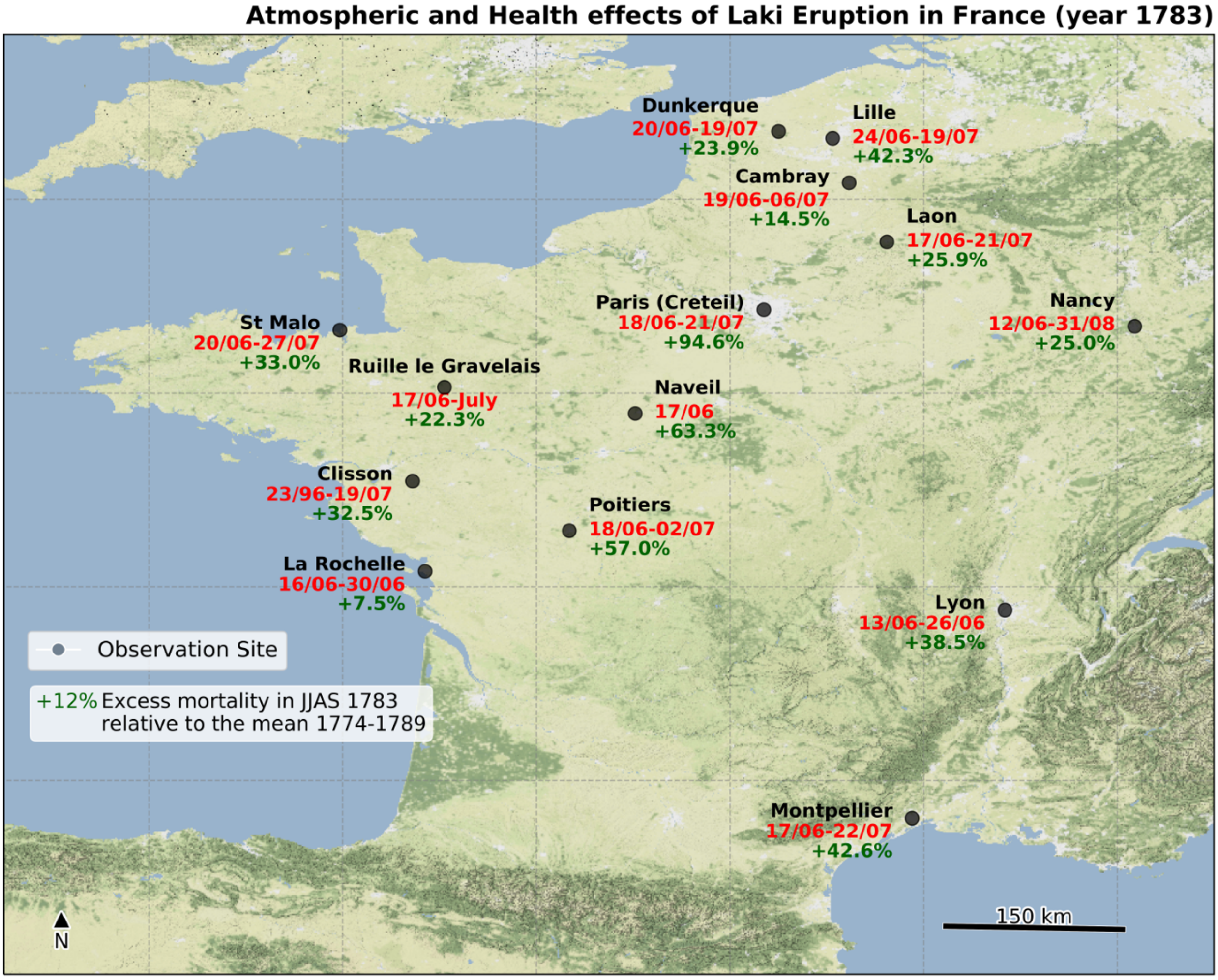

Figure S2

Supplementary Figure S2: Cities for which parishes with registers of Baptism, Weddings and Deceased were analysed to infer the increased mortality of the summer (JJAS) 1783 relative to the 16-years mean (from 1774 to 1789). The dates indicated in red point to periods of frequent fog observation.

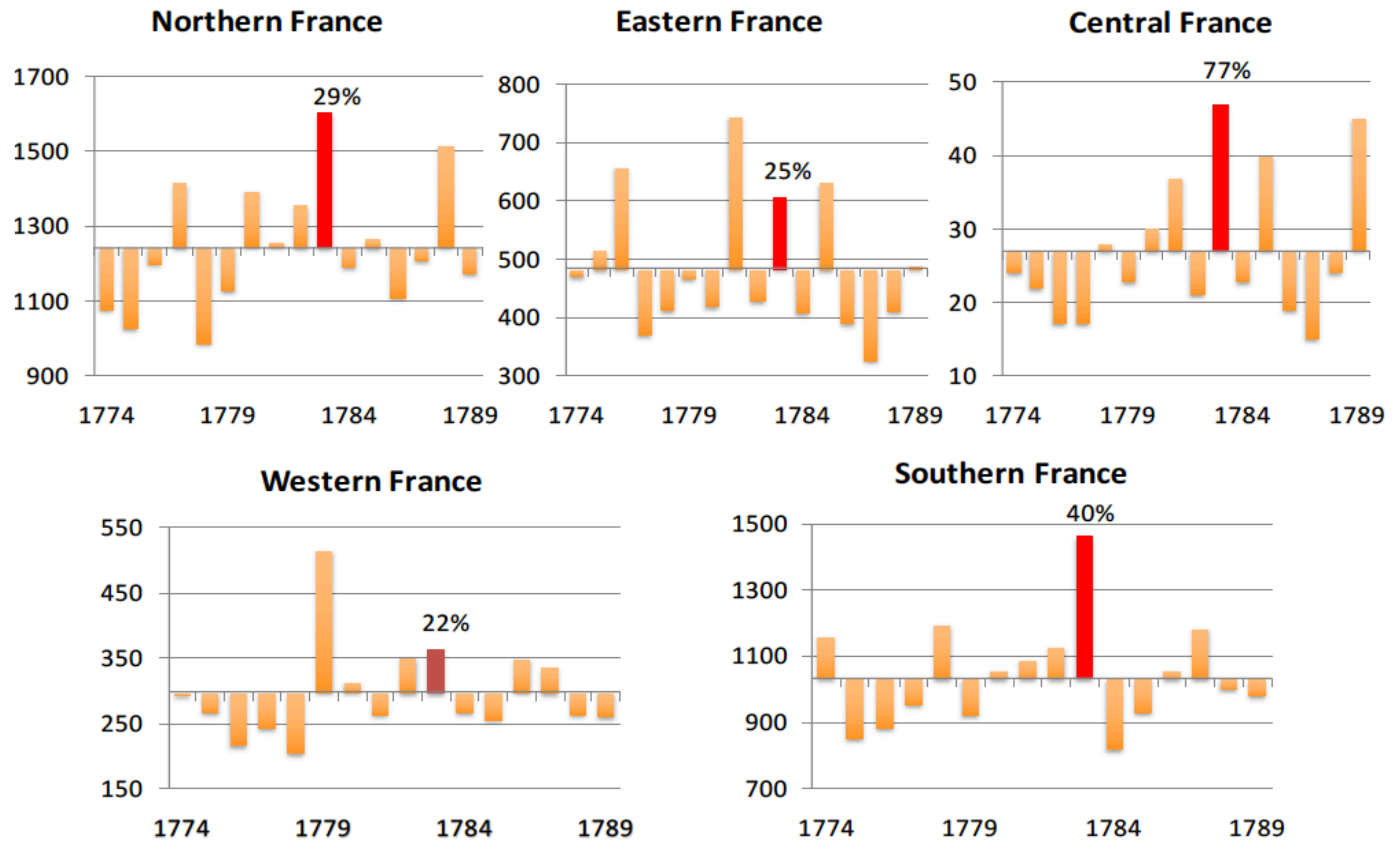

Supplementary Figure S3: Deviation from the mean number of deceased for the period JJAS. The list of parishes is given on Table 3. The column marked in red indicates the JJAS period for 1783.The deviation (%) of the number of deceased from the mean of 15 years is indicated. Note that the different regions have unequal numbers of deceased (Table S3) that correspond to the number and size of the parishes that have been analyse. Respectively, 40, 15, 1, 10 and 34% of the deaths occurred in Northern, Eastern, Central, Western and Southern France.
